# Projecting global shifts in the invasive potential of *Bidens pilosa* L. under climate change using species distribution models

**DOI:** 10.3389/fpls.2025.1580278

**Published:** 2025-05-15

**Authors:** Linran Fan, Chunxiao Mi, Jialu Li, Yanjun Zhang, Haifang Zhang, Guilong Zhang, Hui Wang

**Affiliations:** Agro-Environmental Protection Institute, Ministry of Agriculture and Rural Affair, Tianjin, China

**Keywords:** *Bidens pilosa* L., invasive alien plant, climate change, geographical distribution, maxent

## Abstract

Invasive species pose significant threats to ecosystems by reducing biodiversity, introducing new diseases, and competing with native species for resources. *Bidens pilosa* L., a globally invasive weed originating in tropical America, severely impacts agricultural productivity by infesting 31 economically vital crops across over 40 countries. This study examined the global distribution of *Bidens pilosa* L., under current and future climate scenarios. Using species distribution models and occurrence data, we identified key factors influencing its spread, including temperature, precipitation, and human influence. Our findings suggest a likely decline of suitable habitats in tropical regions and an expansion into temperate regions, with climate suitability decreasing under higher temperatures. Additionally, historical reconstructions emphasize that the rapid spread of the species was facilitated by maritime trade routes. Management strategies are proposed that emphasize the need for enhanced control measures in high-risk areas and conservation efforts in its native range in tropical America. Overall, this research contributes to understanding the dynamics of *B. pilosa* distribution and informs proactive management strategies to mitigate its ecological and economic impacts.

## 1 Introduction

Biological invasion is defined as the process during which a species acquires a competitive advantage following the removal of natural barriers to its proliferation. This advantage facilitates rapid dispersal and the colonization of new niches within recipient ecosystems, ultimately leading to the establishment of dominance (Valéry et al., 2008). They significantly contribute to global environmental change, posing significant threats to biodiversity, ecosystem services, and human well-being (Strayer, 2012; Shackleton et al., 2018; Bongaarts, 2019). As globalization facilitates the movement of species across borders, the ongoing challenge of biological invasions on most continents is expected to continue to increase through 2050 (Pysek et al., 2010; Seebens et al., 2021). Failure to comprehensively address this issue could have profound and lasting consequences for the health and integrity of ecosystems worldwide, emphasizing the critical need for concerted efforts in research, policy, and public awareness initiatives. Therefore, biological invasions will remain a significant global environmental concern in 21st century. Previous studies have shown that among the most worrisome invaders are invasive alien plants (IAPs), which are having increasingly severe impacts on ecosystem dynamics, economic systems, and public health (Subedi et al., 2014; Rai and Singh, 2020; Seebens et al., 2021).

The 2021 Intergovernmental Panel on Climate Change (IPCC) Sixth Assessment Report (AR6) stated that human activities, primarily through emissions of greenhouse gases, have clearly caused global warming, with global surface temperature reaching 1.1°C above 1850–1900 during 2011-2020. This warming trend has impacted the distribution and spread dynamics of IAPs (Hellmann et al., 2008; Flory et al., 2022). Studies have shown that IAPs respond differently to climate change, with some species experiencing range expansion (Osland et al., 2023), while others show a trend toward contraction (Huang et al., 2023), and in some cases, both phenomena occur simultaneously (Wang et al., 2022; Nikkel et al., 2023; Evans et al., 2024). Therefore, it is crucial to understand the complex relationship between climate change and shifts in IAPs distribution. Furthermore, in addition to climatic factors, human activities and soil conditions play an important role in shaping the distribution patterns of IAPs. Human interventions in ecosystems, particularly in the Anthropocene era, not only facilitate invasions but also influence the underlying mechanisms that drive these invasion (Kueffer, 2017). Variations in soil properties such as pH, nutrient availability, and texture can directly impact the establishment, growth, and competitive ability of invasive plants. This highlights the importance of understanding soil-plant interactions for effective management strategies.

Species distribution models (SDMs) are widely utilized in ecological research to explore species-environment relationships (Guisan and Thuiller, 2005). These models have been extensively applied to predict the potential distributions of various invasive species, such as *Phragmites australis*, *Pistia stratiotes*, *Artemisia vulgaris*, *Quercus arkansana*, *Quercus acerifolia*, and *Eriocheir sinensis*, among others (Valéry et al., 2008; Uden et al., 2015; Rodríguez-Rey et al., 2019; Sofaer et al., 2019; Chandra et al., 2023a, b; Zheng et al., 2024). Recognized for its superior predictive ability and minimalist functionality, MaxEnt has been favored over other methods (Phillips et al., 2006; Merow et al., 2013; Radosavljevic and Anderson, 2014; Cobben et al., 2015; Wen et al., 2024), demonstrating better performance compared to alternatives such as BIOCLIM, DOMAIN, and infinite weighted logistic regression (Wisz et al., 2008). Furthermore, MaxEnt can be utilized to provide conservative yet highly accurate estimates of the habitat suitability for invasive species in the landscape (West et al., 2016).

*Bidens pilosa* L. is an annual broad-leaved herbaceous plant belonging to the Asteraceae family. It is native to tropical America and widely distributed in tropical, subtropical, and temperate regions worldwide at present (Pysek et al., 2004; Wu and Wang, 2005). *B. pilosa* is recognized as a troublesome invasive species in over 40 countries worldwide, exerting significant adverse impacts on agricultural productivity and ecosystem integrity. Its pronounced ecological plasticity enables it to thrive across a broad range of habitats, from anthropogenically disturbed sites such as gardens and roadsides to intensively managed agricultural landscapes. Of particular concern is its ability to establish aggressive infestations within 31 crop systems across these countries, notably affecting key staples such as corn, sugarcane, cotton, and rice—crops that underpin global food security and economic resilience (Sharma et al., 2023; Kato-Noguchi and Kurniadie, 2024). *B. pilosa* exhibits numerous adaptive characteristics that enable it to thrive in different environmental conditions, including high seed production, efficient dispersal, wide germination range, and the ability to grow in various soil types. Its rapid growth and resource consumption enable it to compete effectively with associated species (Yue et al., 2019). Besides, its allelopathic properties hinder the growth of other plants (Batish et al., 2002), and make it a potential host for pests and diseases detrimental to crops and native species (Panizzi and Lucini, 2022). The infestation of *B. pilosa* often results in decreased crop production and quality (Blanco et al., 1996; Osland et al., 2023). Enhanced monitoring and prevention efforts are crucial to mitigate further losses caused by *B. pilosa*.

Despite the ecological and agricultural production implications of *B. pilosa*, there remains limited understanding regarding its potential distributional shifts under changing climate conditions. These gaps highlight the necessity for comprehensive, globally focused studies that consider the dynamic interplay between climate change and species invasiveness. Considering future species distributions under various climate change scenarios and management interventions is essential for formulating effective long-term strategies (Richardson et al., 2010). To address this knowledge gap, our study aims to build historical intrusion path, analyze spatiotemporal trends, and identify the key factors influencing the distribution of *B. pilosa*. By assessing areas that will become suitable or unsuitable under future climate scenarios, our research will aid in prioritizing management actions and developing proactive strategies to minimize the impact of *B. pilosa* on natural ecosystems and agriculture.

## 2 Materials and methods

### 2.1 Occurrence data

**Figure 1:**
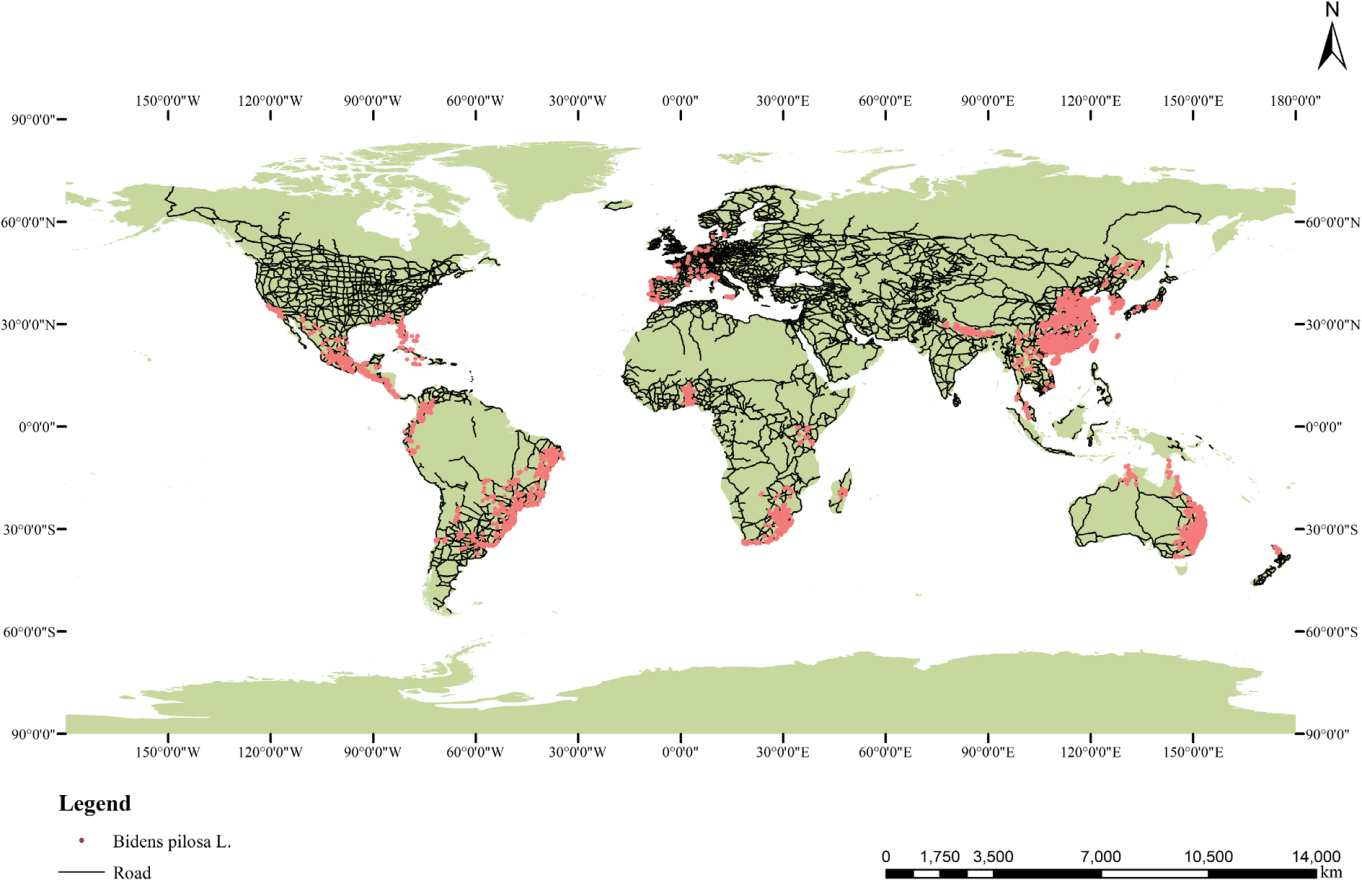
Occurrence of *B. pilosa* across the world.

Most of the distribution records of *B. pilosa* were obtained online, including 41,303 records from the Global Biodiversity Information Facility (GBIF: https://www.gbif.org/occurrence/download?taxon_key=5391845), and 1751 records from the Chinese Virtual Herbarium (https://www.cvh.ac.cn/index.php). There are 326 records of our field survey(During the summer of 2022-2023, we conducted field surveys across nearly all provinces in China where *B. pilosa* has been reported. In total, we collected over 20,000 geospatial data points. After comparing and filtering the data from both years, we obtained the final dataset for analysis.). We obtained a total of 43,380 occurrence records of *Bidens pilosa* L. from worldwide sources. After importing these datasets into ArcGIS 10.2, we filtered out unreliable data and removed points with missing environmental information. In addition, only one occurrence record was retained within each 10 × 10 km raster cell. This process resulted in a final dataset of 12,803 occurrence records globally (**Figure 1**). The longitude and latitude of occurrence records for each continent are provided in **Supplementary Excel Files**.

### 2.2 Predictor variables and climate change scenarios

Drawing on existing research, we selected 56 predictor variables to identify the main factors influencing the distribution pattern of *B. pilosa* worldwide (Yang et al., 2022). These factors include climatic, anthropogenic, and soil variables. Bioclimatic variables, including 19 derived from monthly temperature and precipitation records, along with elevation data, were obtained from the WorldClim 2.1 database (https://www.worldclim.org) at a 2.5-minute resolution. Soil variables were sourced from the Harmonized World Soil Database v1.2 of the United Nations Food and Agriculture Organization (https://www.fao.org/soils-portal/data-hub/soil-maps-and-databases/harmonized-world-soil-database-v12/en/) (Yang et al., 2022). Human footprint (HFP), population (POP), and human influence index (HII) data were acquired from the Center for International Earth Science Information Network (https://sedac.ciesin.columbia.edu/). To address collinearity issues, a Pearson correlation analysis was conducted among the 56 variables, with a correlation coefficient greater than 0.8 being considered a strong correlation (Dormann et al., 2013). Variables with lower contribution to prediction probability were eliminated using the Jackknife method, resulting in 18 predictor variables selected for *B. pilosa* distribution modeling. The 18 predictor variables were classified into three groups: climate variables, human variables, and environmental variables, as presented in **Table 1**.

**Table 1:** Variables used in the potential distribution modeling of *B. pilosa*.

Group	Variable	Description
Climate variables	bio1*	Annual mean temperature
	bio2	Mean monthly temperature range
	bio3*	Isothermality ( (BIO2/BIO7) × 100)
	bio4*	Temperature seasonality (STD × 100)
	bio5	Max temperature of warmest month
	bio6	Min temperature of coldest month
	bio7	Temperature annual range (5–6)
	bio8	Mean temperature of wettest quarter
	bio9	Mean temperature of driest quarter
	bio10*	Mean temperature of warmest quarter
	bio11	Mean temperature of coldest quarter
	bio12	Annual precipitation
	bio13	Precipitation of wettest month
	bio14*	Precipitation of driest month
	bio15	Precipitation seasonality (CV - coefficient of variation)
	bio16	Precipitation of wettest quarter
	bio17*	Precipitation of driest quarter
	bio18*	Precipitation of warmest quarter
	bio19*	Precipitation of coldest quarter
Human variables	HFP*	Human Footprint
	HII*	Human Influence Index
	POP	Population
Environmental variables	T_GRAVEL	Topsoil Graver Content
	T_SAND	Topsoil Sand Fraction
	T_SILT	Topsoil Silt Fraction
	T_CLAY*	Topsoil Clay Fraction
	T_USDA_TEX	Topsoil USDA Texture Classification
	T_REF_BULK*	Topsoil Reference Bulk Density
	T_OC	Topsoil Organic Carbon
	T_PH_H2O*	Topsoil pH (H_2_O)
	T_CEC_CLAY	Topsoil CEC (clay)
	T_CEC_SOIL	Topsoil CEC (soil)
	T_BS	Topsoil Base Saturation
	T_TEB	Topsoil TEB
	T_CACO3	Topsoil Calcium Carbonate
	T_CASO4	Topsoil Gypsum
	T_ESP	Topsoil Sodicity
	T_ECE	Topsoil Salinity
	S_GRAVEL	Subsoil Graver Content
	S_SAND	Subsoil Sand Fraction
	S_SILT*	Subsoil Silt Fraction
	S_CLAY*	Subsoil Clay Fraction
	S_USDA_TEX*	Subsoil USDA Texture Classification
	S_REF_BULK	Subsoil Reference Bulk Density
	S_OC	Subsoil Organic Carbon
	S_PH_H2O	Subsoil pH (H2O)
	S_CEC_CLAY	Subsoil CEC (clay)
	S_CEC_SOIL	Subsoil CEC (soil)
	S_BS	Subsoil Base Saturation
	S_TEB	Subsoil TEB
	S_CACO3	Subsoil Calcium Carbonate
	S_CASO4	Subsoil Gypsum
	S_ESP*	Subsoil Sodicity
	S_ECE	Subsoil Salinity
	Elev*	Altitude

The variables selected for analysis are marked with an asterisk (*).

We utilized future climate projections from the Intergovernmental Panel on Climate Change (IPCC) 6th Assessment Report, which are based on Shared Socioeconomic Pathways (SSPs). The SSPs, intended to span the range of plausible futures, are based on five narratives describing broad socioeconomic trends that could shape future society (Riahi et al., 2017). Taking into account Asia has the most serious invasion of *B. pilosa*, we chose two timelines (2041–2060 and 2081–2100) and four scenarios obtained under the BCC-CSM2-MR model from the WorldClim 2.1 database: SSP126 (sustainability, the most optimistic scenario reflecting RCP2.6 from 5th report), SSP245 (middle of the road, moderate scenario reflecting RCP4.5), SSP370 (regional rivalry, not used in 5th report) and SSP585 (fossil-fuel based development or business-as-usual, reflecting RCP8.5).

### 2.3 Data analysis

The MaxEnt approach is widely used to model the spatial distribution of different species. The basic concept of MaxEnt modeling is to derive the probability distribution with maximum entropy based on occurrence data within given constraints. In contrast to parametric models, MaxEnt was specifically designed to handle presence-only data (Phillips et al., 2006). Since the available data only indicate the presence of the species, we employed the MaxEnt algorithm to construct a species distribution model for *B. pilosa*.

To optimize the model and prevent overfitting, it is crucial to appropriately set relevant parameters. Two key parameters in model calibration are the regularization multiplier (RM) and the combination of features (FCs), both of which are essential for achieving optimal model performance (Muscarella et al., 2014). We used the R package ENMeval to select FC and RM values as optimization parameters for subsequent predictions, with the minimum corrected Akaike Information Criterion (ΔAICc) set to 0 (Warren and Seifert, 2011; Huang et al., 2023). Our results showed that the optimal RM value for Bidens pilosa was 0.5, and the best feature combinations were LQPTH. The distribution records and predictor variables were imported separately into MaxEnt 3.4.1 software. For occurrence data, 10000 background points were randomly selected as pseudo-absences, with 75% of the occurrence records allocated for training and the remaining 25% for testing purposes. The RM was set to 0.5, and the FC was set to LQPTH. To minimize uncertainty, we conducted 10 replicated runs of cross-validation while keeping other settings at their default values. Finally, the average result was considered to represent the potential distribution of *B. pilosa*.

The area under the receiver operating characteristic curve (AUC) was used to assess the accuracy of the model (Baasch et al., 2010). The AUC is calculated by plotting sensitivity against “1-specificity” and ranges from 0 to 1, with values closer to 1 indicating higher prediction accuracy. Prediction ability is rated as failing (0.5–0.6), poor (0.6–0.7), fair (0.7–0.8), good (0.8–0.9), or excellent (0.9–1).

The results from MaxEnt were imported into ArcGIS 10.2 and converted to raster. We reclassified data from various climatic conditions to assess future shifts in suitable habitats under four climate scenarios, compared to historical conditions, using the raster calculator. The potential geographical distribution of *B. pilosa* under current and future climates was classified into the following four categories: unsuitable (P < 0.1), poor (0.1 ≤ P < 0.3), moderate (0.3 ≤ P < 0.5), and high (0.5 ≤ P < 1). The raster-to-point tool was then applied, and the mean distribution centroid was calculated for each climate scenario.

We used ArcGIS 10.2 with the geographic coordinate system GCS_WGS_1984 to map the historical expansion process of *B. pilosa* over per 25-year period worldwide. The occurrences cumulative along the Y-axis.

## 3 Results

### 3.1 Invasive historical reconstruction

**Figure 2:**
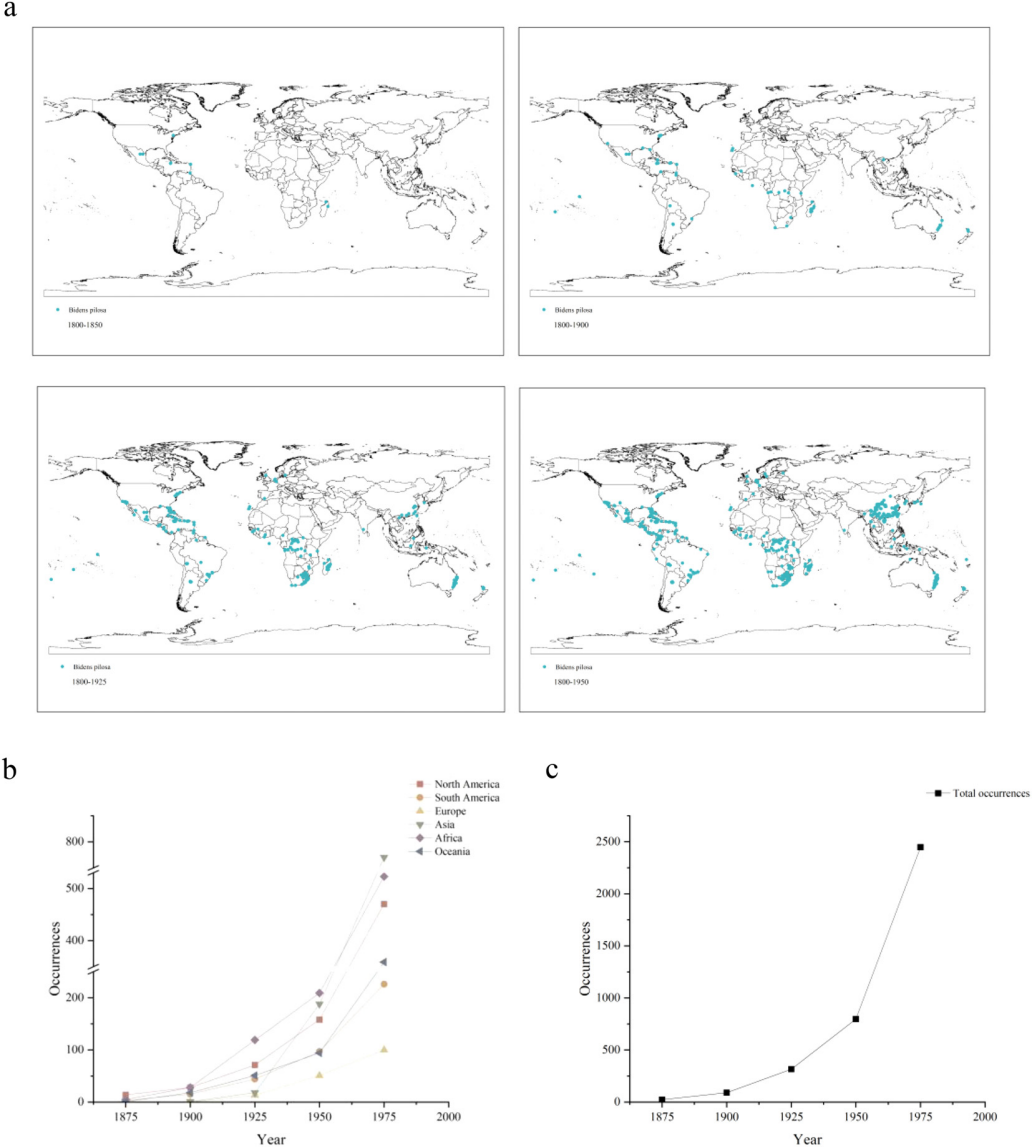
**(a)** Historical reconstruction of the expansion process and increased species occurrence of *B. pilosa* from 1800 to 2000. **(b)** Changes in *B. pilosa* occurrences data across different continents from 1875 to 1975. **(c)** Total occurrences data changes of *B. pilosa* from 1875 to 1975.

**Figure 2** illustrates the historical distribution of *B. pilosa*. Originating from the American continent, its first recorded occurrence outside the Americas dates back to 1847, on the southeastern islands of Africa. Within 50 years, it rapidly expanded to numerous countries across Africa. The species then rapidly expanded its range along coastal regions to Oceania, Asia, and Europe in 1869, 1888, and 1908, respectively. The trends associated with species occurrence from 1875 to 1975 are also shown in **Figure 2**. The spread of *B. pilosa* across continents initially occurred at a slow pace, followed by an explosive increase in population expansion after the establishment of a certain number of sites, reflecting a typical invasion process observed in invasive species. Particularly noteworthy is the exceptionally rapid growth rate observed after spreading to Asia.

### 3.2 Model accuracy evaluation and key predictor variables

**Figure 3:**
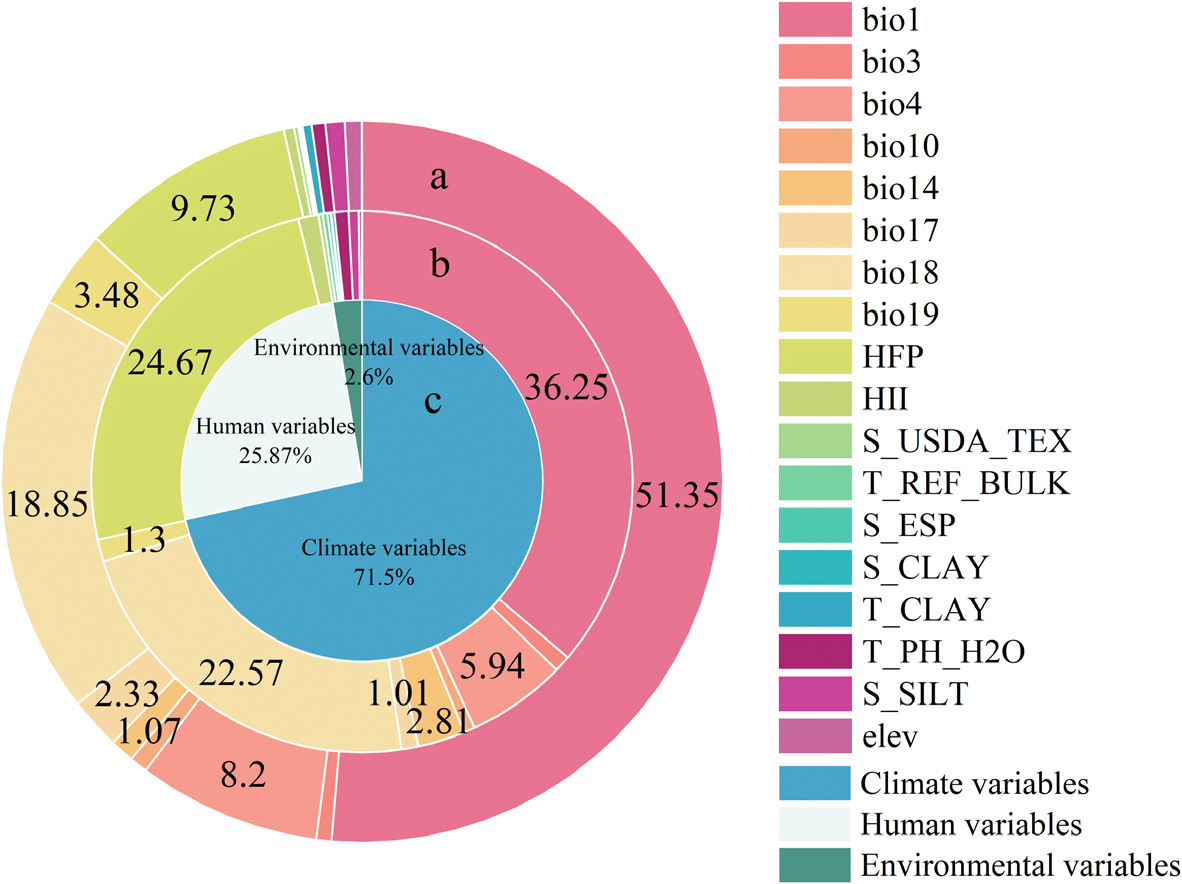
The contributions of different predictor groups and different variables in explaining distribution pattern of *B. pilosa*. **(a)** Permutation importance of all variables calculated by the MaxEnt model. **(b)**Percent contributions of all variables calculated by the MaxEnt model. **(c)** Contributions of different predictor groups calculated by the MaxEnt model.

Our models demonstrated good predictive accuracy, as indicated by an average AUC value of 0.82 across the 10 validation runs. The highest importance was attributed to bio1 (Annual mean temperature, 36.25%), as indicated by the percent contributions of all variables in the model. This was followed by bio18 (Precipitation of the warmest quarter, 22.57%), HFP (Human Footprint, 24.67%), bio4 (Temperature seasonality, 5.94%), and bio14 (Precipitation of the driest month, 2.81%) (**Figure 3**). The contributions of different predictor groups exhibited significant disparities, with the climate variables reaching 71.5%. The contributions of Human variables and Environmental variables are 25.87% and 2.6%, respectively.

### 3.3 Current potential distribution

**Figure 4:**
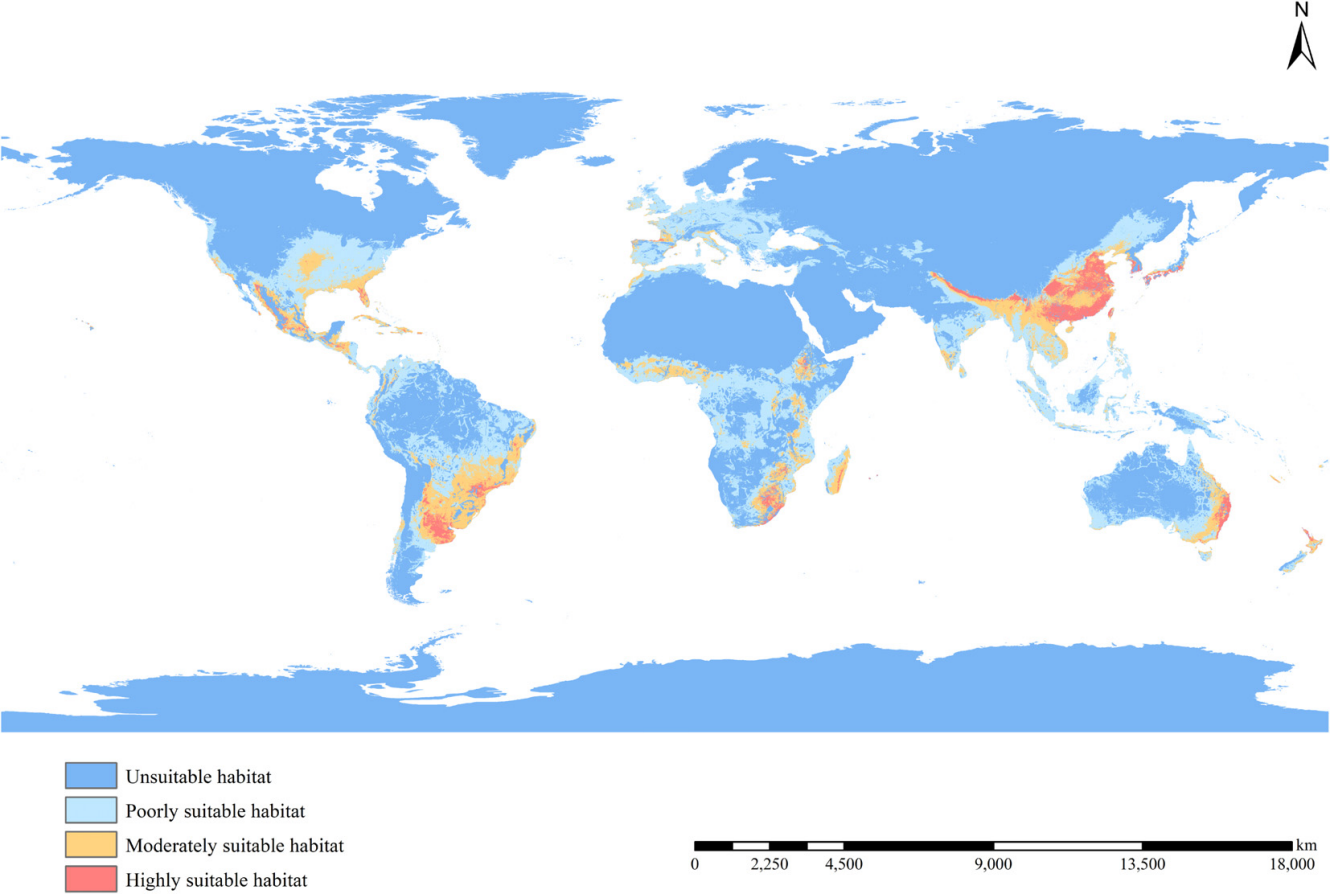
Potential distribution of *B. pilosa* under current climate.

**Figure 5:**
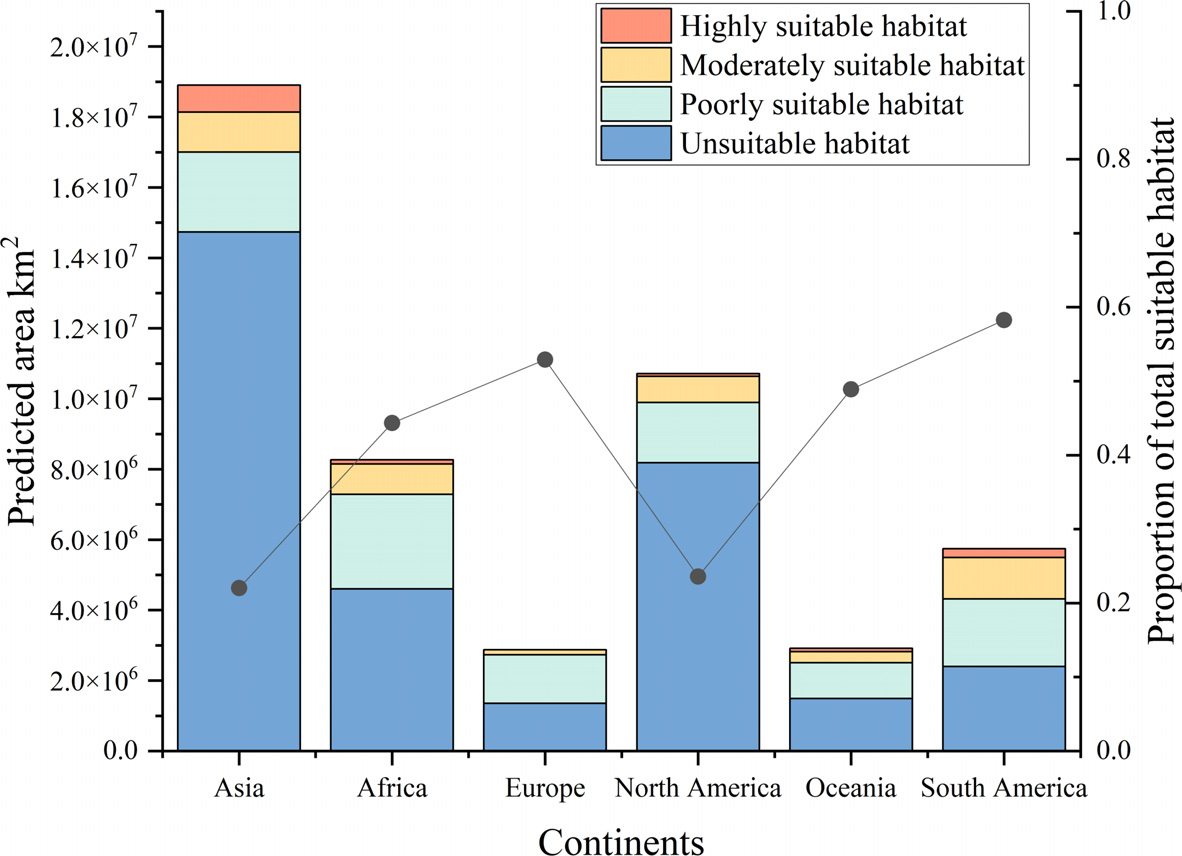
Different types of suitable areas for *B. pilosa* across six continents under the current climate.

**Figure 4** shows the potential distribution of *B. pilosa* under current climatic conditions. Coastal regions have the highest invasion potential, with suitable areas decreasing with distance from the sea. Furthermore, the potential geographic distribution demonstrates a north-south gradient, with the most suitable areas concentrated in the southern mainland. Particularly significant potential distributions are observed in southeastern Asia, eastern Oceania, eastern Africa, southern North America, and southeastern South America. Although many current occurrences of *B. pilosa*, particularly in Southeast Asia and eastern Oceania, are consistent with suitable climates, our model identifies additional areas ripe for invasion, particularly in central and southern Europe, western Oceania, and southwest Africa. The proportion of all suitable habitat areas is, in descending order, South America (58.26%), Europe (52.91%), Oceania (48.89%), Africa (44.34%), North America (23.58%), and Asia (22.04%) (**Figure 5**). Asia in particular has the largest area of particularly suitable habitats (761,000 km^2^).

### 3.4 Future potential distribution under different time periods and climate change scenarios

**Figure 6:**
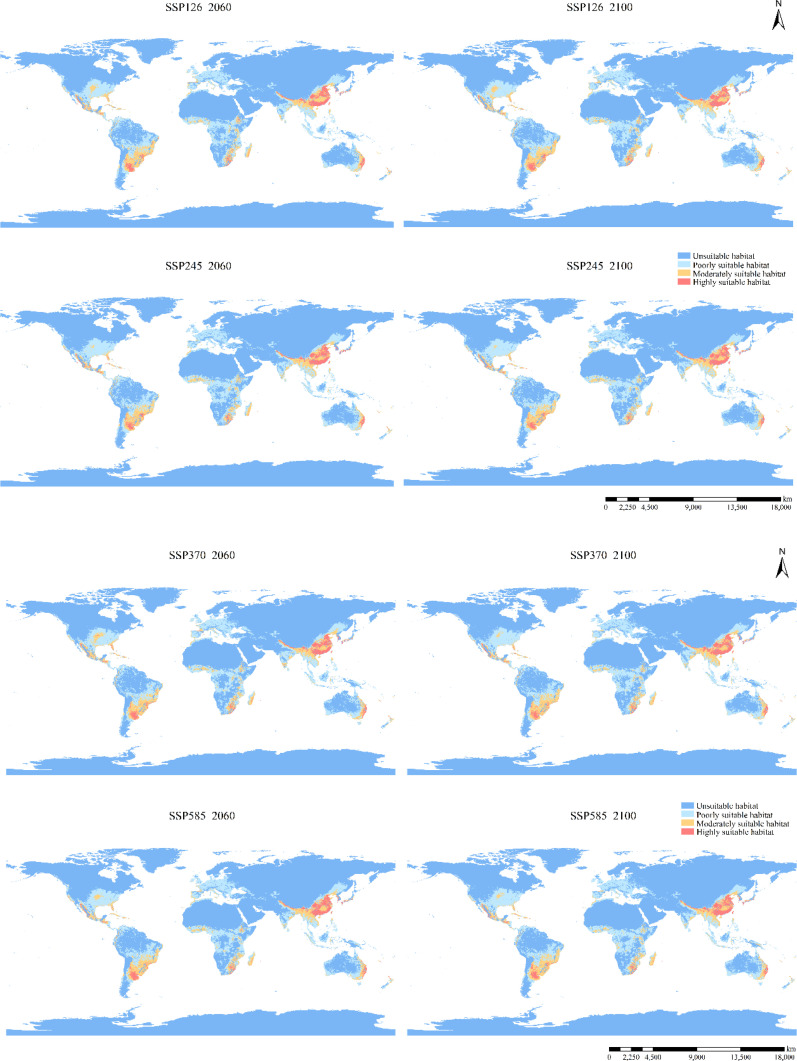
Potential distribution of *B. pilosa* under future climate.

Compared to the current potential distribution, the primary habitat area is projected to remain similar under future climatic conditions (**Figures 4**, **6**). Predictions for the 2041–2060 period in all scenarios indicate range expansion in Argentina, Brazil, Congo, Poland, Ukraine, the northeast of China, and the northwest of the United States. In the period 2081–2100 under the SSP126 scenario, the total expansion area reached 9.04% (**Table 2**), with the main distribution becoming more continuous, primarily concentrated in various European countries. Significant increases are also observed in northern North America and northeastern Asia. Apart from the SSP126 scenario, other predicted results show minor contractions and migration towards colder regions. Overall, as climate change intensifies and time progresses, the changes in distribution are expected to become more pronounced.

**Table 2:** Size of projected range contraction and expansion (related to the predicted current range) of *B. pilosa* for all climate change scenarios and studied timelines.

Timeline	SSP	Total suitable habitat area/km^2^	Range contraction (%)	Range expansion (%)	Total change area/km^2^
Current		1.69×10^7^			
2041-2060	126	1.68×10^7^	8.74	6.59	2.59×10^6^
	245	1.69×10^7^	8.97	7.87	2.84×10^6^
	370	1.68×10^7^	9.31	8.24	2.96×10^6^
	585	1.65×10^7^	11.05	7.95	3.21×10^6^
2081-2100	126	1.73×10^7^	6.81	9.04	2.67×10^6^
	245	1.66×10^7^	12.13	7.71	3.35×10^6^
	370	1.68×10^7^	12.75	11.13	4.03×10^6^
	585	1.67×10^7^	14.14	11.27	4.29×10^6^

**Figure 7:**
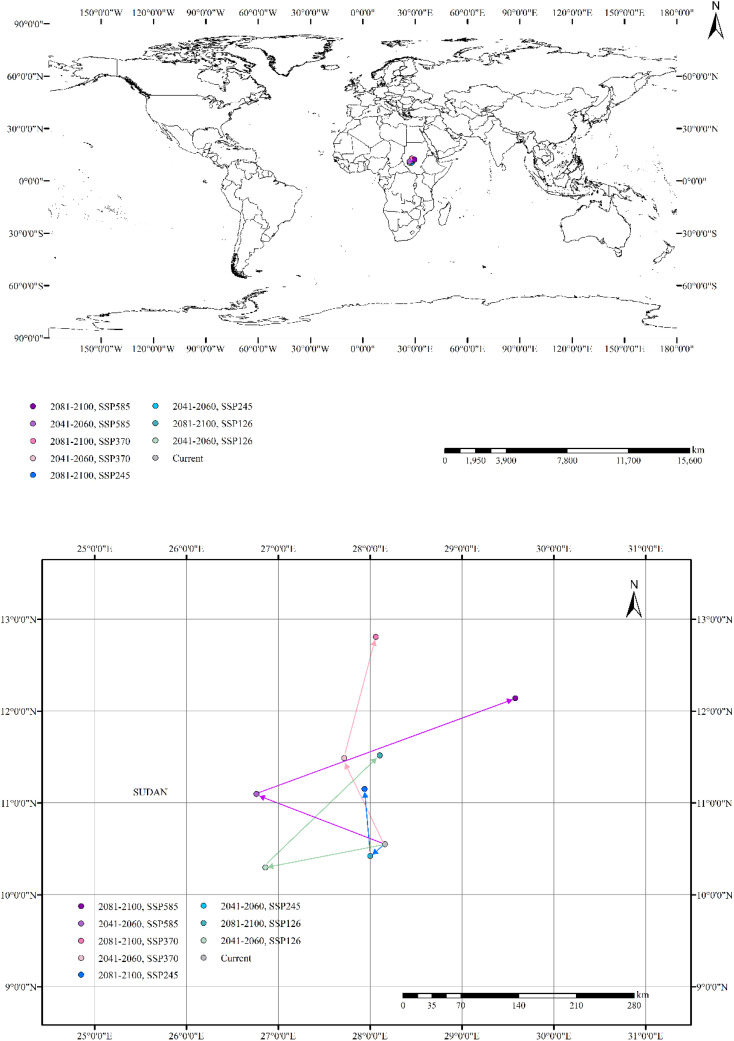
Distribution center shifts of *B. pilosa* under the current and future climate scenarios.

The distribution center of *B. pilosa* was located within Sudan at coordinates (28.160810°E, 10.550958°N) under the current climate. (**Figure 7**). Between 2041 to 2060, the distribution center is projected to shift westward due to climate change. Migration directions varied in different climate scenarios, with centroids shifting towards the southwest under SSP126 and SSP245 scenarios, while showing a stronger shift to the north under SSP370 and SSP585. During the period 2081–2100, the distribution centers is supposed to move towards the northern region. Under the SSP585 scenario, the distribution center of *B. pilosa* is projected to shift more than 200 km north of its current position. Under four climate scenarios, the distribution center of *Bidens pilosa* is likely to shift northward during 2081–2100 compared to the period of 2041-2060. In future scenarios, the distribution center of *B. pilosa* within Sudan is anticipated to shift to higher latitudes or altitudes compared to the current climate.

## 4 Discussion

### 4.1 Invasive historical reconstruction

Invasive species that establish populations in novel environments typically undergo a five-stage process: introduction, colonization, incubation, spread and outbreak (With, 2002; Aikio et al., 2010; Blackburn et al., 2011). Our historical reconstruction of *B. pilosa* invasion is consistent with this evolutionary process. Specifically, the reconstruction revealed a gradual increase in species occurrence over a period of 25 years after the invasion of a new continent, followed by a massive population increase after about 75 years. The lack of significant increases immediately after population invasion could be attributed to a lag phase closely linked to an adaptation period, abrupt invasion mechanisms, and surrounding environmental changes (Crooks and Soule, 1999). Based on invasion history, *B. pilosa* spread to different continents via maritime trade routes. It was initially observed in coastal or island nations such as Madagascar, Vietnam, the United Kingdom, the Netherlands, and New Zealand before gradually expanding its range into the interior of the mainland. The exponential growth of world trade since the late 15th century, evidenced by the increase in shipping tonnage (Fayle, 2005), has led to a corresponding rise in biotic invasions (Mack et al., 2000). In general, maritime trade was an important factor in its spread.

### 4.2 Key predictor variables affecting the distribution of *B. pilosa*

The present study represents an empirical investigation of the invasion potential of *B. pilosa* under current and future climates. Among the predictor variables examined, our findings suggest that temperature, precipitation, and human footprint likely play an important role in regulating the distribution of invasive *B. pilosa*. These results align with recent studies suggesting temperature-related factors as primary determinants of invasive species distribution (Lozano, 2021; Anibaba et al., 2022; Dinh et al., 2022; Yang et al., 2023). *B. pilosa* is an annual herbaceous plant that typically germinates between April and May and flowers from August to September, with a minimum germination temperature requirement exceeding 15°C. Temperature exerts extensive effects on plant growth by influencing metabolic processes such as photosynthesis, respiration, transpiration, as well as synthesis and transportation of organic matter (Qaderi and Reid, 2008; Yamori et al., 2022). Additionally, both the percentage and rate of germination have been observed to decrease (Qaderi and Reid, 2008). Therefore, the changes in annual mean temperature will have a significant impact on the distribution of *B. pilosa*. Furthermore, precipitation during the warmest quarter affects seedling survival rates and nutrient accumulation, influencing reproductive capacity. Previous reports suggest that the plant prefers warmer climates with high precipitation, although it has been shown to be able to withstand a wide range of environmental conditions, from tropical to mild-temperate climates (Sharma et al., 2023). Our results also demonstrate that human activities influence species distribution alongside climatic factors alone (Yang et al., 2023). *Bidens pilosa* produces abundant heteromorphic (central and peripheral) achenes with specialized shapes that can adhere to human clothing and animal fur, making human activities another significant factor in the distribution of these invasive plants. As human influence intensifies, this may accelerate the spread and expansion of IAPs, consistent with previous research (Fang et al., 2021; Yang et al., 2023). This discovery confirms the conclusion from the reconstruction of invasion history, which indicates the spread of *B. pilosa* through human-mediated transportation via sea and land routes in the context of commercial activities.

### 4.3 Differences in distribution changes of *B. pilosa* under future climates

Our study revealed that coastal and riverine regions exhibit a high incidence of *B. pilosa* invasion, a notable observation given the origin from the tropical Americas (Sharma et al., 2023). This species, which favors sunlight and moisture and proliferates rapidly, thrives in habitats with favorable hydrothermal conditions (Wang et al., 2020). Consequently, the warm and humid climate prevalent in middle-low latitude coastal areas makes them especially conducive to its survival. Additionally, the impact of trade flows from coastal ports could contribute to this phenomenon (Seebens et al., 2015).

With the exception of the SSP126 scenario, our models of the potential current range of *B. pilosa* and its future distribution during 2041–2060 and 2081–2100 deviate from the most widely held assumption that climate change will drive range expansion of IAPs (Chauhan et al., 2019; Paz-Dyderska et al., 2021; Yang et al., 2023). In other words, the distribution of invasive plants is changing, but is trending downwards with climate change. Many studies support our results (Anibaba et al., 2022; Mengistu et al., 2023), which indicate variations in dispersal modes, growth habits, and expansion extents between invasive plant species.

While our study predicted a likely decline in climate suitability of *B. pilosa* under future climate scenarios, it also suggests an expansion of suitable climate conditions in colder regions. Several factors contribute to this result: 1) An increase in the average annual temperature reduces the potential distribution area of *B. Pilosa* (Gamar and Qaderi, 2019). The germination rate of *B. pilosa* seeds reached more than 80% under the constant temperature of 15-30°C, but the temperature increases significantly inhibited germination (Hong et al., 2004; Chauhan et al., 2019). In addition, low temperatures can favor the competitive ability of *B. Pilosa* (Yue et al., 2021). Consequently, the increase in annual mean temperature has an adverse effect on the growth and development of *B. pilosa* in tropical regions, while it shows an opposite response in high latitudes. 2) In the future, high carbon dioxide emissions and the frequency of extreme weather events (such as increases in extreme high temperatures, decreases in extreme low temperatures, and increases in intense precipitation events) are expected to decrease the habitable area for *B. pilosa* (Easterling et al., 2000; Bellard et al., 2013). 3) As a C3 plant, *B. pilosa* has a high transpiration coefficient; however, the anticipated climate warming will increase transpiration and soil water losses, which will consequently lead to lower survival rates in tropical regions (Chauhan et al., 2019; Poorter et al., 2022). 4) The expansion of suitable areas has reached saturation, resulting in small fluctuations or contractions. 5) Prediction for the 2081–2100 SSP126 scenario revealed that the suitable area will be expanded. In this scenario consumption is oriented toward low material growth and lower resource and energy intensity. That is, the SSP126 scenario is the only one of the four scenarios in which climate change is in a positive direction. In this scenario, the amount of carbon dioxide emitted from human activities will be reduced, and the rate of global warming will slow down. The downward trend in global warming is conducive to the spread of *B. pilosa*. This conclusion also supports the above reasons from a different angle. Despite these future climate conditions, *B. pilosa* is expected to expand towards colder, higher latitudes.

### 4.4 Future considerations and management of *B. pilosa* in the invaded and native ranges

Our model predicts a likely decline in climatically suitable habitats for *B. pilosa* across most climate scenarios, which is encouraging news for eradication efforts in invaded areas. However, it should be noted that the predicted contraction range is relatively small, and a significant portion of the invasion areas are still under substantial invasion pressure. Therefore, there is an urgent need to enhance management and control measures in high-risk regions. Biological control is emerging as one of the most important approaches to combat invasive plants. Several natural enemies of *B. pilosa* have been documented in its native range, including *Ralstonia solanacearum* Smith, Sonchus yellow net virus, *Cercospora bidentis* Tharp, Bidens mosaic virus, and *Sclerotinia sclerotiorum* (Lib.) de Baary. Nevertheless, current management strategies primarily rely on chemical and physical treatments (Guatimosim et al., 2015; Kato-Noguchi and Kurniadie, 2024). These methods include mechanical mowing as well as herbicide application using glyphosate atrazine 2-4-D glyphosate imazethapyr metribuzin paraquat (Chauhan et al., 2019). The extensive use of glyphosate not only destroys the ecosystem, but also accumulates in animals through bioaccumulation and spreads along the food chain, producing toxic effects on non - target organisms. Therefore, it is important to intensify research efforts to find further sustainable and effective solutions, such as biological control, to contain the spread of *B. pilosa*.

For highly suitable habitats, a combination of physical and chemical methods should be used to control and prevent the spread. For moderately and poorly suitable habitats, we should actively promote scientific knowledge about *B. pilosa* and establish public awareness regarding invasive species prevention and control. As our model predicts expansion of *B. pilosa* into colder regions, early detection systems should be established in Argentina, Brazil, Congo, Poland, Ukraine, northeast China, and northwest United States. We recommend establishing priority management areas at open ports in these regions.

In our model, we predicted the persistence of *B. pilosa* in its native range, tropical America. It has no advantages in its native habitats and our projections show that only a small proportion of suitable acreage across the Americas (**Figure 2**). Therefore, conservation efforts should prioritize managing and preserving *B. pilosa* within its native range in the tropical Americas to safeguard native biodiversity, despite the challenges posed by its invasive nature.

### 4.5 Study limitations

Our study focused exclusively on environmental variables and human influence. Although both anthropogenic and environmental factors jointly shape invasion dynamics, the distinct climatic signal identified in our results (**Figure 3**) necessitates treating these variables separately to better isolate the impacts of global warming from local habitat modulators. However, it is important to note that plant invasions are also influenced by biotic interactions. For instance, the stiff awns of Bidens pilosa can adhere to the bodies of animals—including birds and mammals—facilitating long-distance dispersal. This animal-mediated seed dispersal may significantly contribute to the invasion process, a factor not explicitly incorporated into our models. Future research should integrate these biotic interactions, particularly the role of birds and other animals in seed dispersal, to further refine predictions of invasion dynamics and improve our understanding of species range expansion (Wang and Wan, 2021).

Furthermore, while species distribution models (SDMs) offer valuable insights into areas with potentially suitable climatic conditions, they do not necessarily indicate successful establishment. Successful invasions depend on a range of ecological processes beyond climate, and overestimation of potential distributions remains a recognized challenge in SDM-based studies.

Lastly, our analysis may be affected by sampling bias. Historical invasion reconstructions are often compromised by incomplete survey techniques and inconsistent monitoring efforts (Delisle et al., 2003). The increasing awareness and documentation of invasive alien plants (IAPs) over time may lead to a progressive rise in recorded occurrences, which might not accurately reflect true expansion dynamics. Future studies should consider employing standardized long-term monitoring and species-specific detection protocols to mitigate these biases.

## Data Availability

The original contributions presented in the study are included in the article/**Supplementary Material**. Further inquiries can be directed to the corresponding authors.
